# **Initiating and Documenting Goals of Care Discussion in Patients with Advanced Pancreatic and Colorectal Cancers**: **A Quality Improvement Project in a Low Resource Setting**

**DOI:** 10.1177/26892820251392545

**Published:** 2025-11-04

**Authors:** Daniel Raj Joseph Thangasamy, Meenakshi V. Venketeswaran, Thendral Ramasamy, Vinutha Suresh, Rathipriya S., Robert Louis A., Ramakrishnan Ayloor Seshadri

**Affiliations:** ^1^ Department of Anaesthesia, Pain, and Palliative Care, Cancer Institute (WIA), Adyar, Chennai, India.; ^2^ Department of Medical Oncology, Cancer Institute (WIA), Adyar, Chennai, India.; ^3^ Department of Surgical Oncology, Integrated Cancer Care Group, Chennai, India

**Keywords:** quality improvement, goals of care, oncology, palliative care, patient care planning, quality of life

## Abstract

Patients with advanced malignancies often end up receiving aggressive interventions which are not aligned with their preferences especially during terminal stages. Discussing and documenting the goals of care (GOC) early in the course of the illness will help patients receive interventions based on their preferences. In resource-constrained settings such as our institution, a regional cancer center in India, a lower-middle socioeconomic country, this also enables effective utilization of life-saving equipment and Intensive care unit care. Since we did not have a standard GOC discussion and documentation process, we initiated a quality improvement (QI) project. This QI initiative was done using the A3 methodology between September 2023 to May 2024 and it was followed up till March 2025. For this project, we chose patients with advanced pancreatic and colorectal cancer with a life expectancy of less than one year. We followed various steps of the QI project such as defining the problem, setting SMART goals, process mapping, root cause analysis, identifying key drivers and interventions to solve the problem, and also planned sustainability measures. As a result of this QI project, we were able to increase the rates of GOC documentation from 0% to 92% in patients with advanced pancreatic and colorectal cancer. Root cause analysis revealed that the absence of a standard operating procedure/document and limited awareness about GOC were the main barriers for which we derived key drivers and interventions. Though creating awareness help to increase the number of patients referred for GOC discussion, our goal was achieved after we created a color-coded GOC document. Introducing a standardized, color-coded GOC documentation process through a QI initiative significantly improved discussion and documentation rates in patients with advanced cancer. Such QI initiatives are feasible in low- and middle-income settings and help align care with patient preferences and save resources.

## Introduction

Patients with advanced malignancies can have anticipated or unanticipated medical emergencies in their disease trajectory for which they may receive aggressive, potentially inappropriate life-sustaining treatment toward the end of life (EOL).^1^ Hence, it is ideal to have a discussion regarding the goals of care (GOC) early in the course of the disease, where the physician elicits and understands the goals, preferences, and values of the patient.^2–4^ These discussions enable not only the patients and caregivers to have a clear care plan but also help the health care team in managing the patients and caregivers during difficult emergency situations.^4^ However, undocumented discussions or difficulty in accessing such documentation coupled with the stressful environment in an emergency room may often result in the emergency or intensive care physician, who is often not part of the patient’s primary care team, to provide treatments that do not align with the wishes of the patient.^4,5^

Palliative care services in India have steadily grown over the past three decades. Although there are more than 800 specialized palliative care services available across the country, the median coverage of palliative care services in India is only around 0.6/100,000 inhabitants.^6,7^ There are gaps in the provision of palliative care services across various cancer centers in the country with many falling short of meeting the essential quality standards.^7,8^ India ranks low in the quality of death index because of various reasons including cultural taboos on discussions about death and dying and lack of advance care planning.^9^ Although in 2018 the Supreme Court of India recognized advance medical directives as essential to uphold the right to a dignified life, empowering patients to express their preferences for EOL care and enabling physicians to act lawfully in alignment with these directives,^10^ GOC discussions are rarely held until very late in the course of disease in India, leading to inappropriate utilization of resources in the intensive care unit (ICU).^2^ While a minimum standard tool for evaluation of palliative care services exists in India, it does not include GOC discussion as either an essential or desirable feature.^11^

Our institution does not have a systematic program of GOC discussion or documentation in patients with advanced malignancies being treated with a palliative intent. Isolated discussions happen between oncologists and patients or their caregivers and there is poor documentation of the same. This has often led to delayed initiation of EOL care and frequent hospital/ICU admissions toward. In a resource-constrained setting such as ours where allocation of valuable resources is of paramount importance, providing potentially inappropriate care for such patients in the hospital or the ICU often deprives more deserving patients of such life-saving measures. Hence, we initiated a quality improvement (QI) project to develop a policy for systematic GOC discussion and documentation in patients with advanced malignancies receiving palliative treatments.

## Methods

This single-center QI project was initiated jointly by the Departments of Pain and Palliative care (PPC), Medical and Surgical Oncology at the Cancer Institute (WIA) in Chennai, India, which is a 550-bedded not-for-profit exclusive tertiary cancer hospital catering to patients across the socioeconomic spectrum. The project was part of the National Cancer Grid of India-Enable Quality Improve Patient care India program (2023–2024 cohort), which is a mentored training program in QI in cancer care using the A3-QI-methodology.^12,13^ The A3 methodology for QI, with its origins in the Toyota automobile industry, is based on the Plan-Do-Study-Act (PDSA) cycle and facilitates consensus building within the team by documenting the project on an A3-sized paper (later modified to fit into a PowerPoint slide). It uses sequential steps to define the problem, set goals, map current processes, analyze root causes of the problem, identify key drivers, derive, and activate interventions to solve the problem and initiate sustainability measures.^13,14^ This QI project ran in parallel with another one, which aimed at improving early referrals of patients with advanced gastrointestinal cancers to palliative services in our institution.^15^

The physicians involved in the project included four palliative care physicians and residents, two surgical and two medical oncologists. Since this was a QI project, the Institutional Ethics Committee exempted it from a formal approval.

The SMART (Specific, Measurable, Attainable, Relevant and Timely) goal of the project was to increase the rate of documentation of GOC discussion in patients with advanced pancreatic and colorectal cancers having oncologist assessed life expectancy of less than one year, discussed in multidisciplinary tumor board and planned for palliative treatment from 0% to 20% between September 2023 to May 2024. Based on this, we targeted the following categories of patients:

1. Patients with metastatic pancreatic cancer planned for first line of chemotherapy or those who are not fit for any disease directed therapy
2. Patients with metastatic colorectal cancer planned for second line of chemotherapy or those who are not fit for any disease directed therapy

### Measures

The outcome measure for our QI project was the percentage of documentation of GOC discussion among patients previously identified as eligible for the same in the multidisciplinary team (MDT) meeting. A time frame of four weeks to have the GOC discussion completed and documented was chosen in order to allow the PPC team to develop a therapeutic relationship with the patient and/or caregiver before having this difficult and sensitive conversation.

The process measures included the number of patients referred by the oncologists for GOC discussion and the percentage of patients for whom GOC discussion was initiated by the palliative care team.

The balancing measures included the additional time spent by patients at the PPC and the average time spent by health care professionals with the patients for GOC discussion.

### Current state and root cause analysis

**FIG. 1.**
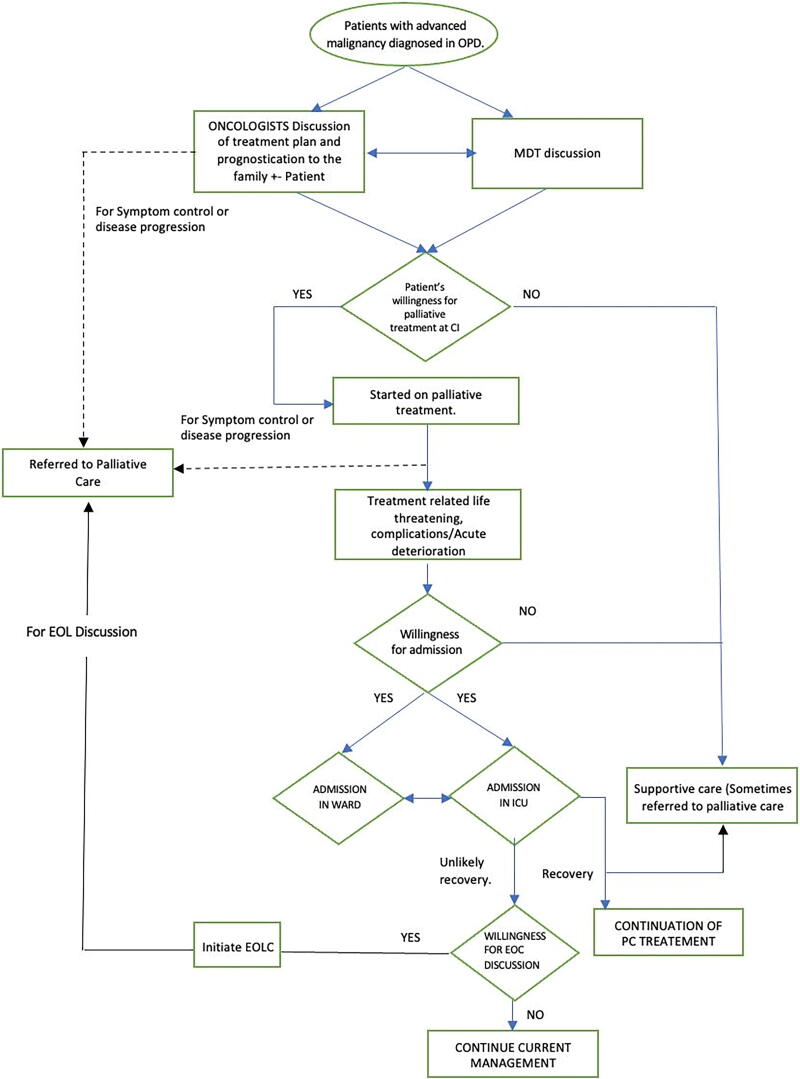
Process map.

A process mapping was done for a current state analysis by shadowing random patients with newly diagnosed advanced colorectal or pancreatic cancer in the out-patient oncology and palliative care clinics during a one-week period (Fig. 1). In addition, we also went through the records of patients with advanced colorectal or pancreatic cancers either admitted to the ICU or for whom a call for EOL care was given to the palliative care team during this week to see if there was documentation of any prior GOC discussion that had taken place with them. Briefly, patients are seen by oncologists in the out-patient clinics and treatment is planned in a multidisciplinary tumor board meeting in which palliative care physicians are not present. In patients with advanced cancers or those receiving palliative intent treatment, early referral to PPC was happening infrequently, GOC discussions did not take place systematically and were not documented in the patient record. Calls to the PPC team for EOL discussion usually happened in the last few days of life after the patient was already admitted in the ICU or on life support.

**FIG. 2.**
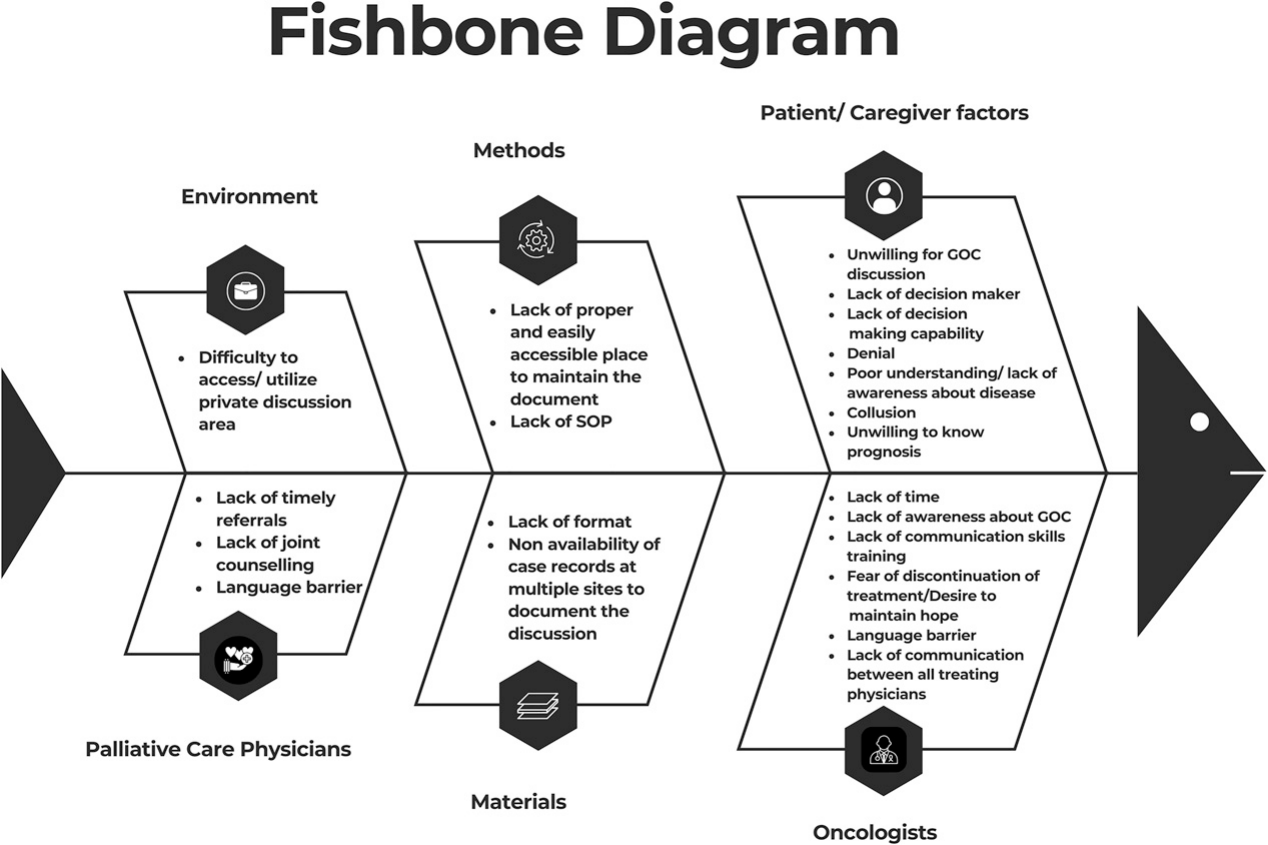
Fishbone diagram.

**FIG. 3.**
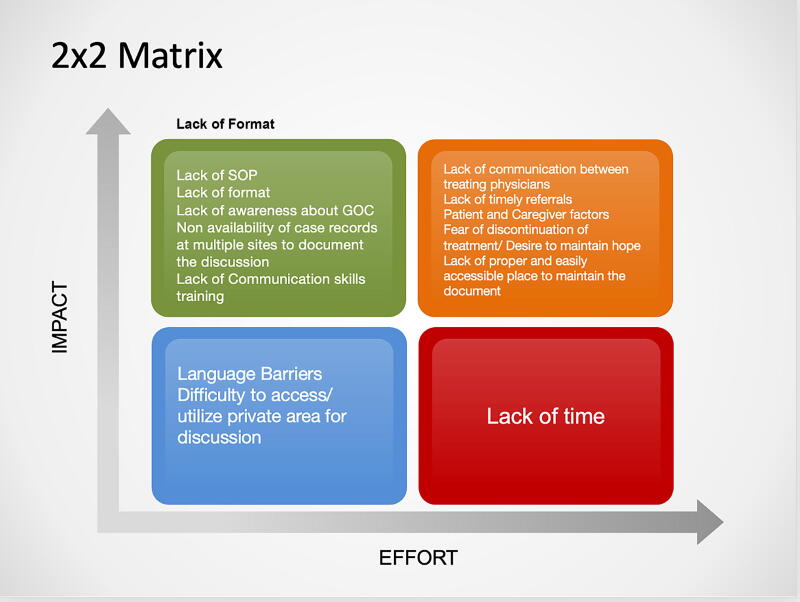
Impact effort matrix.

We identified the root causes for not having a GOC discussion (Fig. 2) by conducting an interview with all the stakeholders (consultants) of both the Gastrointestinal oncology and PPC team individually and collated them using the “5 Why’s” tool.^14^ The stakeholders then rated them on a scale of 1–10 with 1 being the least likely cause for not having this discussion and 10 being the most likely. A Pareto analysis was done to identify the vital few root causes based on this rating. The Pareto principle is the observation that for many outcomes, roughly 80% of the effects or results come from 20% of the causes.^16^ However, in our project, since the Pareto was not contributory, we performed a 2 × 2 impact effort analysis (Fig. 3) to identify the high-impact low-effort causes which could be focused upon to achieve the SMART goal. These included the nonavailability of a standard operating procedure for GOC and documentation template in our institution, lack of awareness about GOC discussion among some oncologists, nonavailability of case records at multiple sites to document the discussion, lack of formal communication skills training, poor coordination between treating physicians and the PPC team, and poor timeliness of PPC referrals.

### Key drivers and interventions

**FIG. 4.**
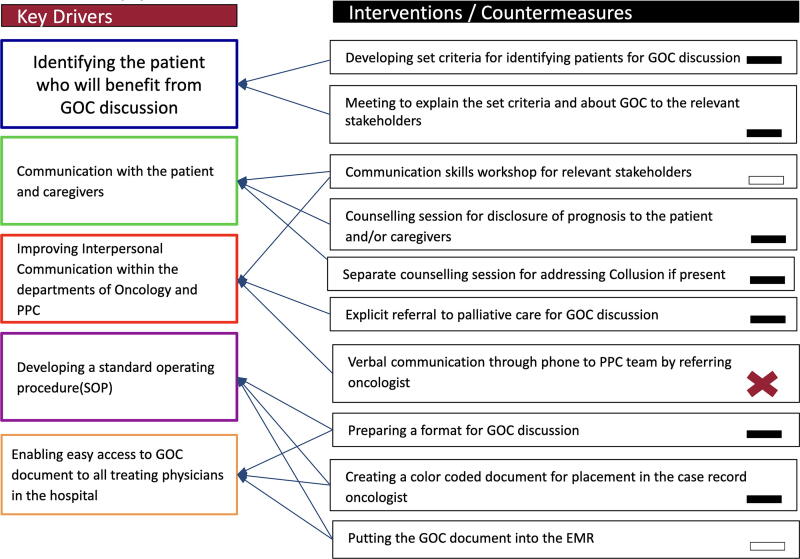
Key drivers and interventions.

Based on the impact-effort matrix we derived the key drivers, devised and implemented various interventions to address the vital few causes (Fig. 4). Some of the key drivers include formulating a standard operating procedure, enabling easy access to the GOC document and identification of the patient for GOC discussion. The interventions included developing criteria for identifying eligible patients; meetings to discuss and explain the criteria, standard operating procedure and the importance of GOC discussion to oncologists; creating a GOC document (Supplementary Data S1) to record the discussions and color coding the same. During the duration of the project some of the interventions were modified, some aborted, and some new interventions were developed based on the PDSA cycle.

**FIG. 5.**
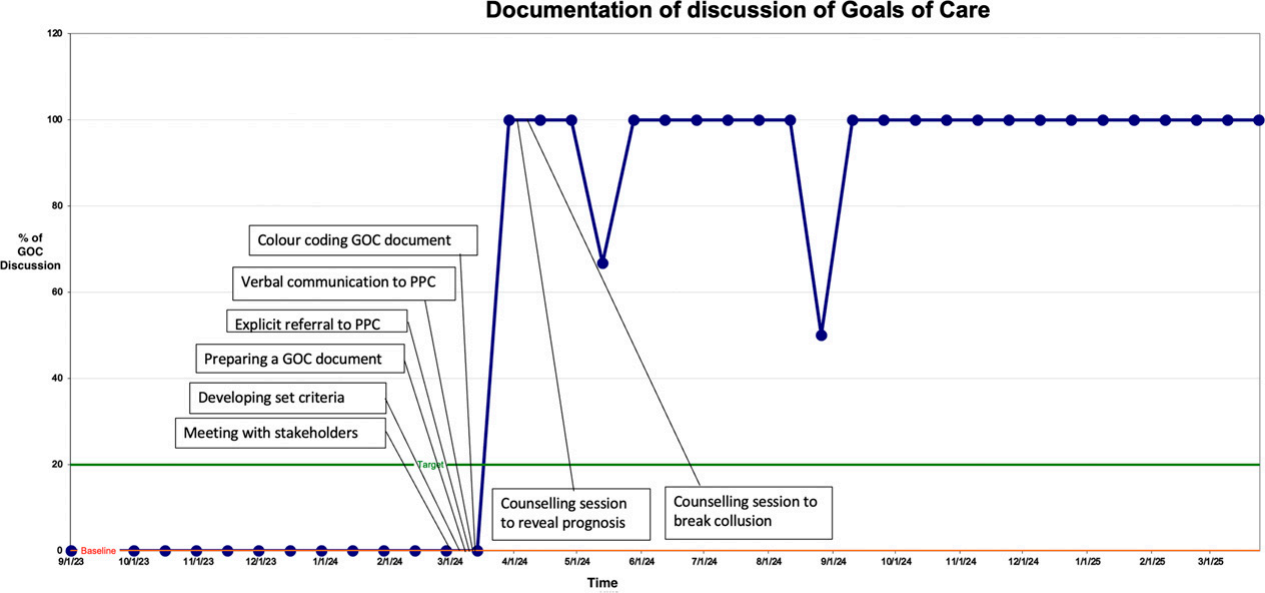
Run chart.

We maintained a run chart to track the progress of the QI project during the entire period of the project. This depicts the time on the X-axis and proportion of eligible patients having a documented GOC discussion on the Y-axis along with the baseline and target limits (Fig. 5). It also shows how the proportion was affected by introducing various interventions at different time points. The entire QI project is represented on a single PowerPoint slide (Supplementary Data S2).

## Results

We identified 26 eligible patients during the period of the project in the MDT meetings who were then referred to the PPC team for GOC discussion. Of these, we were able to complete GOC discussion and document the same in 24 patients. In two patients the discussion took place without documentation of the same. Through this QI project, we were able to increase the rates of GOC documentation from 0% to 92% in patients with advanced pancreatic and colorectal cancer over a period of one year.

Initially, our run chart remained at zero despite receiving referrals for GOC discussions since the interventions for documentation were yet to be put in place (Fig. 5). Once the standard operating procedure was established, a purple color chart was created for documenting the GOC discussion, following which there was a steep rise in the run chart. The purple color was chosen for its uniqueness and easy identification in the patient’s medical chart.

One of the interventions-verbal communication to the PPC team by the referring oncologist did not work for us and hence this was aborted. This was attributed to the fact that the oncology and palliative care outpatient clinics are not located adjacent to each other and sometimes the PPC team were not reachable over phone while the referral happens. Hence, we modified the GOC document to enable the referring oncologist to record the information given to the patient and/or caregiver about the disease prognosis.

Some interventions such as a communication workshop for the stakeholders and developing a digital version of the GOC document into the electronic medical records could not be implemented, the former due to the inability to schedule a mutually convenient time to conduct a workshop during this time period and the latter due to the imminent transition to the new electronic medical records system.

There was a decline in the documentation during the month of May 2024, probably due to the fact that the palliative care team was away for a conference and hence could not complete the discussion. The run chart also saw a decline in the month of August 2024 since there was a change in the constitution of the GI team, and referrals to PPC from the MDT reduced. We realized that the oncology residents, who see a large number of patients in the clinics rotate through the gastrointestinal oncology unit every three months and that every new batch were ignorant of this project. Therefore, we conducted meetings with the new batch of residents to educate them about the QI project and the importance of GOC discussions.

**FIG. 6.**
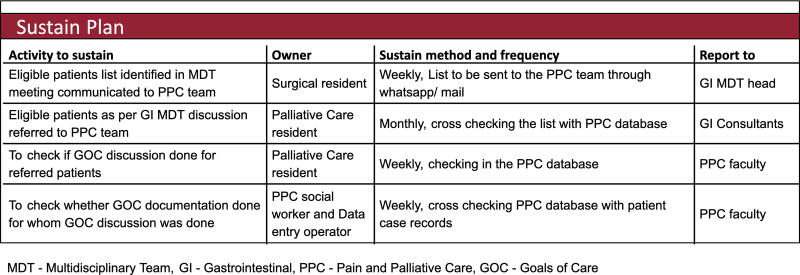
Sustainability plan.

We developed sustainability plans to ensure continuation of GOC discussion and documentation beyond the life of this project (Fig. 6). The future plan was to incorporate this entire process in the new electronic medical records platform.

As a consequence of this QI project, an additional 176 patients, many with malignancies other than advanced pancreatic/colorectal cancer were referred for GOC discussions. Among these, GOC documentation was complete in 65 patients, while GOC discussions were initiated in the others.

## Discussion

This QI project led to an improvement of GOC discussions in the target population from 0% to 92%. The success of this project could be attributed to good mentoring, following an established methodology and a teamwork involving physicians from multiple concerned disciplines and the parallel QI project aimed at increasing early referrals of patients with advanced cancer for palliative care.^15^ Other QI studies on GOC documentation have reported improvement of documentation rates ranging from 13% to 31%.^17,18^

Documenting GOC discussions has been identified as an important component of quality care in oncology^19–21^ and failure to or inadequate documentation has been shown to result in patients receiving interventions that were not in alignment with the patient preferences.^4,5^

The lack of a standard protocol and a template for documentation, which were identified as a major barrier in our study, was also reported in similar QI projects from high-income countries^17,18,22^ Another root cause identified in our study—the lack of awareness about GOC discussions and of communication skills among oncologists to initiate/conduct these sensitive discussions—was also reported by other studies from India and Texas.^2,23^ The perception among oncologists in our institution that patients may not be willing for GOC discussion, either because that will make them lose hope and abandon treatment, was yet another reason for not having a GOC discussion. A similar barrier was reported in a study from Canada.^24^ All these highlight the fact that there are some universal barriers for GOC documentation that cut across geographic or sociocultural contexts.

However, any intervention in a QI project must be contextually relevant. While a GOC discussion is ideally conducted by the primary physician/oncologist or in their presence^25,26^ this was not feasible in our institution. The oncology and PPC outpatient units are located in the different buildings, and each oncologist sees around 50–60 patients daily. GOC discussions require privacy and considerable time. Many times, these discussions span several days especially in our country, where the patient or primary caregiver may not be the decision maker. Hence, we decided that the oncologist would explain the prognosis and initiate the GOC discussion and then refer the patient to the PPC team who would then have a detailed discussion and document the same. E-mail reminders were introduced in some QI projects to alert the physicians to do GOC discussion and document the code status for all the eligible patients.^17,18^ Instead, due to the limited capacity of our electronic medical records software and the imminent transition to a new one, the oncologist inserted the purple-colored GOC template document into the medical charts of patients identified during the MDT meeting to alert the PPC physicians to initiate GOC discussion. However, we realized that in emergency situations, the emergency room physician may not be aware if a GOC discussion had taken place earlier. Hence, we designed a purple sticker which could be pasted on the patient handbook—a booklet which the patient carries that has a hospital identification number and a short record of disease status and treatment. This sticker would alert the physician to seek out the GOC document from the medical charts.

QI is an ongoing process in which the interventions evolve as we go through multiple PDSA cycles to achieve our SMART goal.^27,28^ Initially, we designed two forms, one with few details of the discussion by the oncology team and the second that is filled by the PPC physician after interaction with the patient and family. This was combined into one for ease of use and also incorporated details to be communicated to the PPC team by the oncologist obviating the need for phone calls between departments given the time constraints of a busy oncology OPD. The forms were created with checkboxes rather than free text to make it user-friendly.

The encouraging results from this QI project led us to offer GOC discussions to patients with advanced cancer of other sites. We hope to apply the learning, experience and process developed through this QI project to all patients with advanced cancers being treated at our institution, so documentation of GOC discussion becomes a standard practice. We designed the GOC discussion template in such a way that it can be easily adapted to all cancer subtypes and hope to incorporate it into the new electronic medical records software. This step highlights the importance of generalizability and sustainability that need to be taken into account while doing a QI project.

### Strengths

Our QI project was designed in a way that it can be easily adapted across the other cancer types in the hospital though we have done it only for advanced pancreatic and colorectal cancer patients. We have created sustainability plans to ensure continuity of documentation of GOC discussions even after the QI project ends. This format could be easily adapted in other institutions especially in the resource-constrained settings.

### Limitations

Though our rates of documentation increased, we did not study if this translated into providing treatment according to their documented preferences. This needs a longer follow-up of the patients than the limited duration of our QI project. Another limitation is that although the documentation of GOC discussion is available in our hospital records, we did not plan to provide patients and/or caregivers a copy of the document, which would be useful if they visit any other hospital in an emergency. However, we will start to give a copy of this document to the patient in the near future. This QI project is unique to the context of our institution. While some barriers may be common to other institutions irrespective of resource constraints, some others may be different, and hence the interventions designed by us may not uniformly apply to other centers.

## Conclusion

The rates of GOC discussion and documentation, an important marker of the quality of care in oncology, can be successfully improved by a quality improvement process involving all the stakeholders. This will help provide treatment towards the EOL according to the patient’s wishes avoiding potentially inappropriate treatment.

## Abbreviations Used

EOL: end of life
GOC: goals of care
ICU: intensive care unit
MDT: Multidisciplinary team
PPC: pain and palliative care
QI: quality improvement
