# Supplementary material for: Initiating and Documenting Goals of Care Discussion in Patients with Advanced Pancreatic and Colorectal Cancers: A Quality Improvement Project in a Low Resource Setting
Source: Palliat Med Rep. 2025 Nov 4;6(1):554–63. doi: 10.1177/26892820251392545 (PMC12670709; doi:10.1177/26892820251392545)
Supplement: Supplementary Data S1 [file 26892820251392545_supplementary_data_s1.docx]

**Goals of Care Discussion(GOC) Document**

**Patient name – Date & Time –**

**Age/Sex - UHID – PPC no –**

**Diagnosis -**

*(Please note – Nos. 1 to 4 to be filled by the primary physician/referring oncologist)*

1. **Awareness about Disease:**

**Patient - Diagnosis Prognosis Collusion Does not want to know**

**Family - Diagnosis Prognosis**

1. **Intent of treatment:**

**Palliative systemic therapy Best supportive care End of life care**

1. **Reason for referral informed to Caregiver Patient**
2. **GOC discussion initiated Yes No, If Yes with Caregiver Patient**

***Signature of the Primary Physician/***

***Referring Oncologist***

*(Please note – This section needs to be filled by the Palliative Care Team/ Physician having GOC discussion when patient/family is ready or during early signs of deterioration/treatment failure)*

**Patient and/or Family present –**

**Caregiver/s -**

**Surrogate Decision maker/s -**

**Discussion Content:**

***Understanding of the disease trajectory and prognosis:***

*By the patient –*

*By the family –*

*Patient’s wishes and expectations –*

*Family’s wishes and expectations –*

**Discussion Outcome:**

*1)Symptoms Management(e.g. addressing pain, Shortness of breath)*

*2)Medical Interventions(e.g. Imaging, Antibiotics, IV fluids, TPN)*

*3)Life Sustaining measures(e.g. Mechanical ventilation, dialysis)*

*4)Cardio pulmonary resuscitation(CPR) note*

**I consent to use this data for publications/scientific presentations Yes No**

**Revisiting Goals of care – Needed / Not needed**

**Done on –**

**Members of the team present –**

**If Revisited, date of original discussion –**

Name & Signature of the patient with Date

Name & Signature of the Doctor with Date

Name & Signature of the Caregiver/s with Date

***(Note : This is not a legal document. This is the preference of the patient/caregiver(s) and it needs to be confirmed again in case of emergency)***

**GOC discussion outcome**

**Place of care Home Hospital ward Hospital ICU Hospice**

**Goals of care Aggressive care Symptom management only**

**CPR Yes No**

**Ventilator Yes No**

**O2 support Yes No**

**Feeding tubes Yes No**

**IV Fluids Yes No**
