# Supplementary figures and images for: Initiating and Documenting Goals of Care Discussion in Patients with Advanced Pancreatic and Colorectal Cancers: A Quality Improvement Project in a Low Resource Setting
Source: Palliat Med Rep. 2025 Nov 4;6(1):554–63. doi: 10.1177/26892820251392545 (PMC12670709; doi:10.1177/26892820251392545)

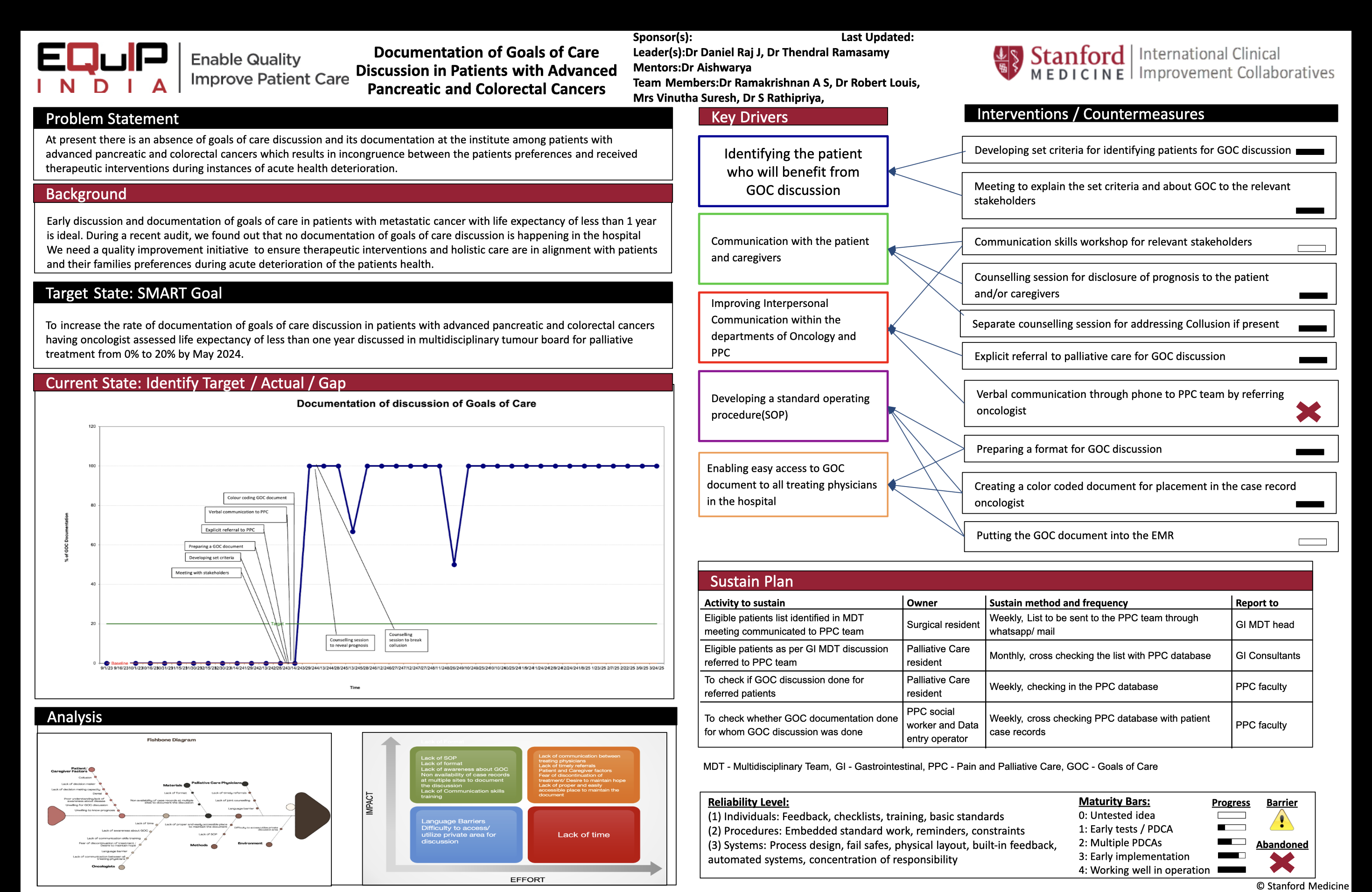

Supplement: Supplementary Data S1 [file 26892820251392545_supplementary_data_s2.tif]
